# Supplementary material for: Mitochondrial stress causes neuronal dysfunction via an ATF4-dependent increase in L-2-hydroxyglutarate
Source: J Cell Biol. 2019 Oct 23;218(12):4007–16. doi: 10.1083/jcb.201904148 (PMC6891100; doi:10.1083/jcb.201904148)
Supplement: Supplemental Materials (PDF) [file JCB_201904148_sm.pdf]

Supplemental material

Hunt et al., https://doi.org/10.1083/jcb.201904148

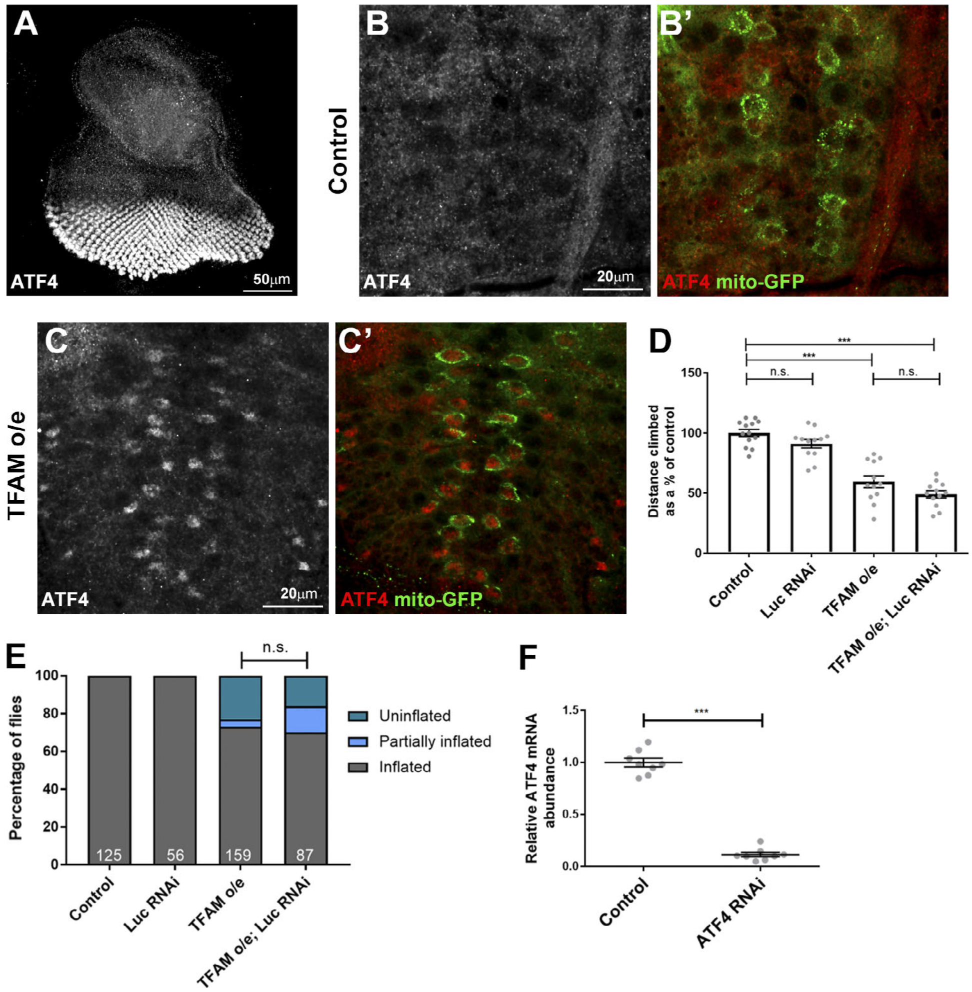

Figure S1. **ATF4 expression, control climbing and wing inflation assays and ATF4 qRT-PCR. (A)** Late third instar larval eye-antennal imaginal disc stained for ATF4 expression. **(B–C′)** ATF4 is not expressed in control larval neurons (B and B′), but its expression is activated in motor neurons overexpressing TFAM (C and C′) using *D42-Gal4*. ATF4 in white in B and C and red in B′ and C′. CD8-GFP (green) marks motor neurons. **(D)** An RNAi targeted against luciferase does not modify the climbing phenotype caused by TFAM overexpression in neurons using *nSyb-Gal4*. Control *n* = 13, Luc RNAi *n* = 12, TFAM overexpression *n* = 12, TFAM overexpression + Luc RNAi *n* = 12. **(E)** An RNAi targeted against luciferase does not modify the wing inflation phenotype caused by TFAM overexpression caused in neurons using *nSyb-Gal4*. Numbers of flies are shown in white. **(F)** qRT-PCR analysis of ATF4 expression in control and ubiquitous ATF4 knockdown larvae using *Da-Gal4*. Control *n* = 8, TFAM overexpression *n* = 8. Controls are *Gal4* hemizygotes. Data are represented as mean ± SEM. n.s., not significant; ***, P ≤ 0.001.

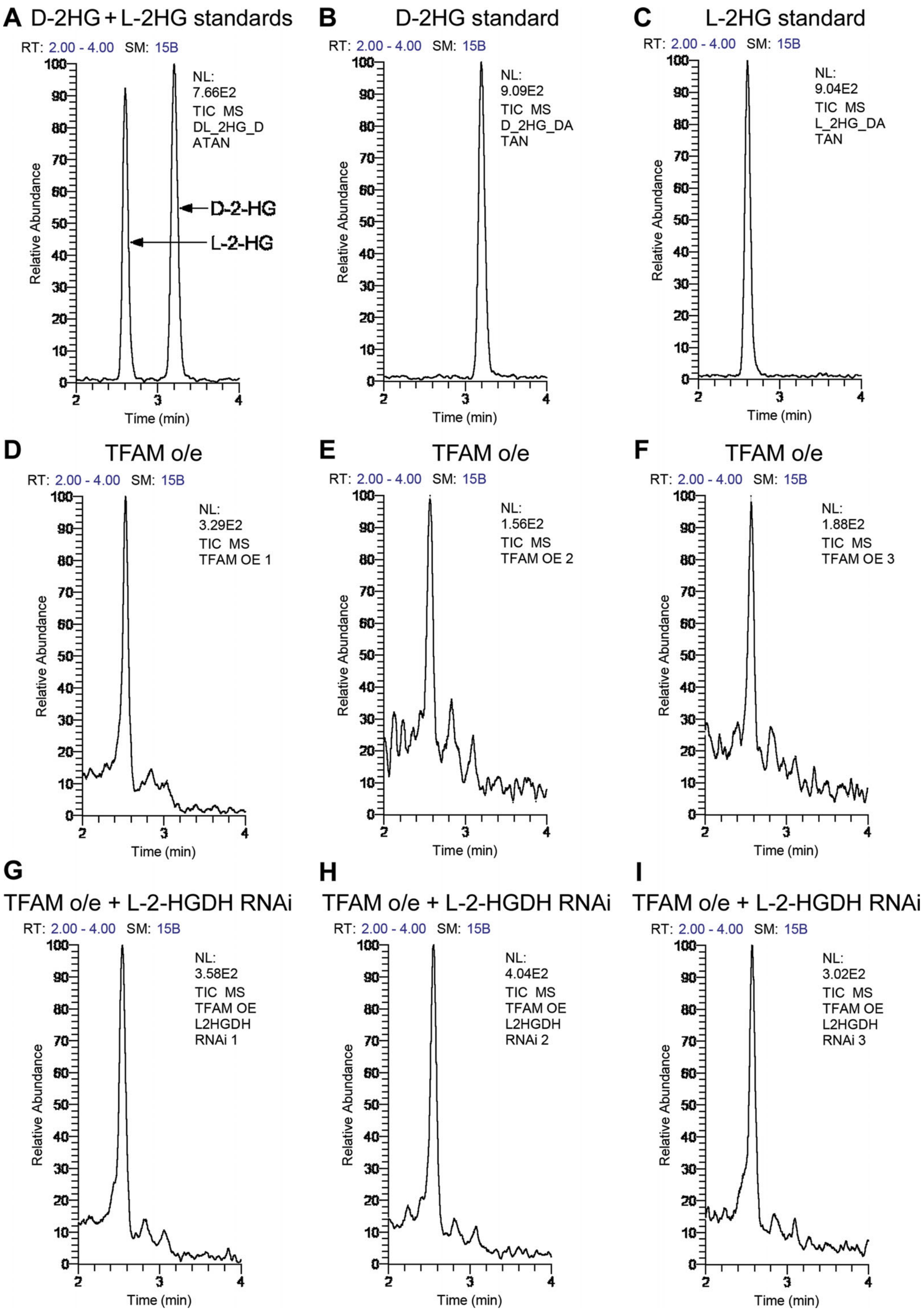

Figure S2. **2-HG measured in heads from flies with pan-neuronal TFAM overexpression is predominantly the L enantiomer.** HPLC-MS/MS was used to determine the enantiomers of 2-HG present. **(A)** Derivitization of a racemic mixture of standards of D and L enantiomers of 2-HG with DATAN allows separation of 2-HG enantiomer derivatives by HPLC retention time. **(B)** Derivitization of D-2-HG standard with DATAN. **(C)** Derivitization of L-2-HG standard with DATAN. **(D–F)** DATAN derivitization of 2-HG extracted from heads from flies overexpressing TFAM with *nSyb-Gal4* (*n* = 3) shows that the majority of 2-HG present is the L-2-HG enantiomer, based on the retention time (compare D–F to C). **(G–I)** DATAN derivitization of 2-HG extracted from flies overexpressing TFAM and L-2-HGDH RNAi with *nSyb-Gal4* (*n* = 3) shows that the majority of 2-HG present is the L-2-HG enantiomer, based on the retention time (compare G–I to C). In D–I, HPLC-MS/MS was performed using independent biological replicates, shown in each panel.

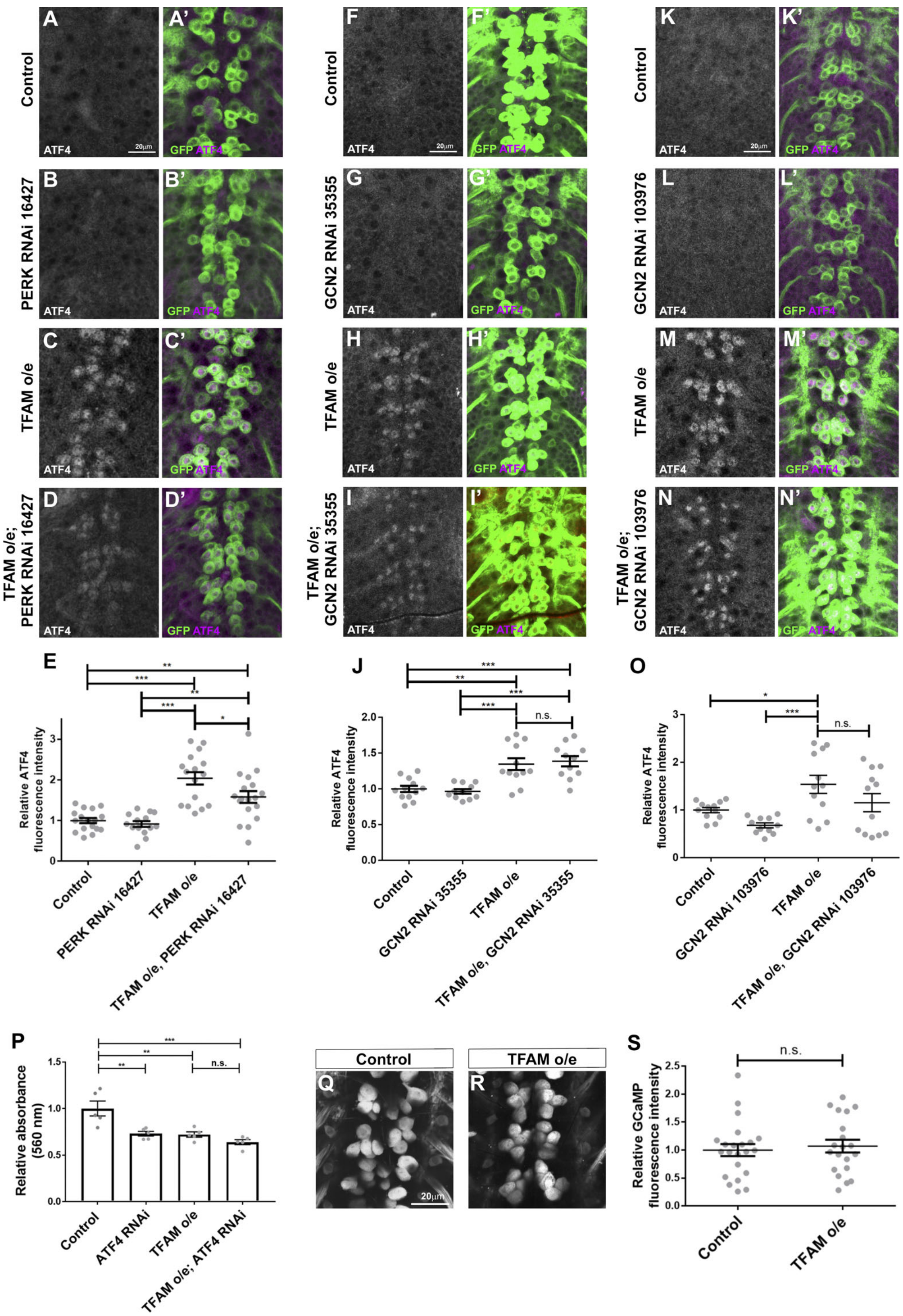

Figure S3. **Mitochondrial stress–induced ATF4 activation requires PERK but not GCN2. (A–D′)** Knockdown of PERK (using RNAi line 16427) prevents the increase in ATF4 expression (white in A–D and magenta in A′–D′) caused by overexpression of TFAM in larval motor neurons using *OK371-Gal4*. **(E)** Quantification of ATF4 expression. Control *n* = 17, PERK RNAi *n* = 14, TFAM overexpression *n* = 16, TFAM overexpression + PERK RNAi *n* = 18. **(F–I′ and K–N′)** Knockdown of GCN2 using two independent RNAi lines (35355 and 103976) does not prevent the increase in ATF4 expression (white in F–N and magenta in F′–N′) caused by overexpression of TFAM in larval motor neurons using *OK371-Gal4*. CD8-GFP expression (green) marks motor neuron cell bodies. **(J)** Quantification of ATF4 expression. Control *n* = 12, GCN2 RNAi 35355 *n* = 11, TFAM overexpression *n* = 12,TFAM overexpression + GCN2 RNAi 35355 *n* = 11. **(O)** Quantification of ATF4 expression. Control *n* = 12, GCN2 RNAi 103976 *n* = 11, TFAM overexpression *n* = 12, TFAM overexpression + GCN2 RNAi 103976 *n* = 12. **(P)** Pan-neuronal overexpression of TFAM using *nSyb-Gal4* reduces hydrogen peroxide levels (determined using Amplex red) in adult heads. TFAM overexpression combined with ATF4 knockdown causes a similar reduction in hydrogen peroxide levels. *n* = 5 for all genotypes. **(Q–S)** GCaMP6m fluorescence in control (Q) or TFAM overexpressing (R) larval motor neurons cell bodies. **(S)** Quantification of GCaMP6m fluorescence in cell bodies, *n* = 22 for control, 20 for TFAM overexpression. Motor neuron expression was using *OK371-Gal4*. Controls are *Gal4* hemizygotes. Data are represented as mean ± SEM. n.s., not significant; *, P ≤ 0.05; **, P ≤ 0.01; ***, P ≤ 0.001.

Table S7. **Details of *Drosophila* lines**

Stock	Source and stock number	Construct ID
w^{1118}	BDSC 6326	-
w;D42-Gal4*	BDSC 8816	-
w;Da-Gal4*	BDSC 55851	-
w^{1118};*OK371-Gal4*	BDSC 26160	-
nSyb-Gal4	From Rita Sousa-Nunes	-
nSyb-Gal4, TFAM^{c01716}	*TFAM*^{c01716} BDSC 10713	-
UAS-Dcr2;OK371-Gal4,UAS-CD8-GFP	From Darren Williams	-
w^{1118};*OK371-Gal4,UAS-nlacz*	*UAS-nlacZ* BDSC 3955	-
w^{1118};*UAS-TFAM3M*	Cagin et al., 2015	-
w^{1118};*UAS-L2HGHD*	Li et al., 2017	-
y^1,v^1;*UAS-L2HGHD dsRNA*	BDSC 51870	HMC03444
y^1,v^1;*UAS-Luciferase dsRNA*	BDSC 31603	JF01355
w^{1118};*OK371-Gal4, UAS-GCAMP6m*	*UAS-GCAMP6m (II) attP40* from BDSC 42748	-
w;UAS-dTrpA1 (III) attP2*	BDSC 26264	-
w^{1118}; *UAS-PERK dsRNA*	VDRC 16427	GD5584
y^1,v^1;*UAS-PERK dsRNA*	BDSC 42499	HMJ02063
y^1, *sc**,v^1;*UAS-GCN2 dsRNA*	BDSC 35355	GL00267
UAS-GCN2 dsRNA	VDRC 103976	KK103566
y^1,v^1;*UAS-ATF4 dsRNA*	BDSC 25985	JF02007
w;UAS-XBP1-EGFP (II)*	BDSC 60730	-
UAS-ATF4-HA	FlyORF F000106	P000084

BDSC, Bloomington Drosophila Stock Center; VDRC, Vienna Drosophila Resource Center; -, not applicable.

Provided online are six tables in Excel. Table S1 shows genes that are significantly misregulated by pan-neuronal (nSyb-Gal4) TFAM overexpression compared to control in larval CNS tissue. Table S2 shows genes that are significantly misregulated by pan-neuronal (nSyb-Gal4) ATF4 overexpression compared to control in larval CNS tissue. Table S3 shows genes that are significantly misregulated in both TFAM overexpression compared to control and ATF4 compared to control conditions. Table S4 shows genes that are significantly misregulated by pan-neuronal (nSyb-Gal4) TFAM overexpression compared to TFAM overexpression combined with ATF4 knockdown in larval CNS tissue. Table S5 shows genes that are significantly misregulated in both TFAM overexpression compared to control and TFAM overexpression compared to TFAM overexpression combined with ATF4 knockdown conditions. Table S6 shows metabolomic analysis of adult heads from flies with pan-neuronal (nSyb-Gal4) TFAM overexpression, ATF4 knockdown, or TFAM overexpression together with ATF4 knockdown and controls.

References

Cagin, U., O.F. Duncan, A.P. Gatt, M.S. Dionne, S.T. Sweeney, and J.M. Bateman. 2015. Mitochondrial retrograde signaling regulates neuronal function. *Proc. Natl. Acad. Sci. USA.* 112:E6000–E6009. https://doi.org/10.1073/pnas.1505036112

Li, H., G. Chawla, A.J. Hurlburt, M.C. Sterrett, O. Zaslaver, J. Cox, J.A. Karty, A.P. Rosebrock, A.A. Caudy, and J.M. Tennessen. 2017. Drosophila larvae synthesize the putative oncometabolite L-2-hydroxyglutarate during normal developmental growth. *Proc. Natl. Acad. Sci. USA.* 114:1353–1358. https://doi.org/10.1073/pnas.1614102114
